# Investigation of Polymers as Matrix Materials for Application in Colorimetric Gas Sensors for the Detection of Ammonia

**DOI:** 10.3390/s25092829

**Published:** 2025-04-30

**Authors:** Sonja Hoffmann, Michael Henfling, Sabine Trupp

**Affiliations:** 1 Fraunhofer Institute for Electronic Microsystems and Solid State Technologies EMFT, 80686 Munich, Germany; 2 Institute of Physics, Bundeswehr University Munich, 85577 Neubiberg, Germany

**Keywords:** colorimetric gas sensor, sensor material, polymer, sensor matrix, ammonia, pH indicators, camera sensor

## Abstract

Colorimetric gas sensors are based on a color changing reaction of a sensor dye upon exposure to an analyte. For most sensor applications, the sensor dye must be immobilized in a sensor matrix. The choice of matrix significantly influences the dye’s response due to different physical and chemical effects. Ideal matrix materials should be transparent, stable, compatible with the sensor dye, and processable. Polymers are often applied as matrix materials, as they can be easily applied to sensor structures. In this study, we present a method to examine the impact of polymers of different structures and functionalities on sensor dyes. Therefore, 18 polymers are studied in combination with the pH indicator bromocresol green regarding their sensitivity to ammonia. The measurement setup is based on a camera as a detector of the color changing reaction of the sensor materials and allows for the simultaneous measurement of the sensor materials. Furthermore, the response and regeneration time, the stability, and the influence of the environmental parameters humidity and temperature on the colorimetric reaction are investigated. The study demonstrates that polymers as sensor matrices have an influence on the response of sensor dyes, due to their different properties, such as polarity. This has to be considered when choosing a suitable sensor matrix.

## 1. Introduction

Colorimetric gas sensors detect analyte gases through a visible color change in a gas-sensitive material, referred to as the sensor material [1]. It typically comprises three essential components: a sensor material, a light source, and a detector that reads out the color change. The fundamental principle involves a color change in the sensor dye upon exposure to the analyte gas. The sensor dye immobilized by a sensor matrix is called the sensor material.

By now, a variety of organic and inorganic matrix materials for colorimetric gas sensors have been reported in the literature, such as silica [2,3], aluminum oxide [4], silicone [5,6], and polymers [7,8]. An important advantage of the application of polymers as sensor matrices is that there is a wide selection of soluble polymers. Solubility in solvents is also critical for processing the sensor material. This property enables easy application, using a variety of coating methods like ink-jet printing [8], screen printing [9], or spin or spray coating [10,11]. Furthermore, polymers have versatile properties due to their structural and functional variety. For application as a sensor matrix, polymers must fulfill several requirements: they must be optically transparent, permeable to the analyte gas, and capable of dissolving the sensor dye. Furthermore, polymers must be mechanically and chemically stable, ensuring that they do not undergo aging or degradation under conditions such as elevated temperatures or exposure to light irradiation [12,13].

Common examples of polymers used as sensor matrices include ethyl cellulose [14], polyvinyl chloride [15], polystyrene [15], and polymethyl methacrylate [14]. The interaction between the polymer, the sensor dye, and the analyte gas is determined by solubility and diffusion properties, described based on the permeability of the material. Permeability P is defined as the product of solubility S and diffusion D [16]:

P = D × S

Permeability depends on the polymer’s morphology and physical characteristics, including crystallinity, molecular orientation, cross-linking, molecular weight, and density. Additionally, factors such as temperature, humidity, and the presence of additives (e.g., plasticizers) can influence permeability and overall sensor performance.

Several studies have explored the impact of polymers as sensor matrices on sensor sensitivity and selectivity for colorimetric gas sensors. For example, Apostilidis et al. demonstrated a combinatorial approach for the development of sensor materials. He investigated the combination of 16 polymers, e.g., ethyl cellulose, cellulose acetate, polyvinyl chloride with several sensor dyes for the detection of oxygen, carbon dioxide, and ammonia to assess sensitivity and cross-sensitivity to humidity and temperature using UV/Vis spectroscopy [15]. Similarly, Courbat et al. compared ethyl cellulose, polymethyl methacrylate, and polyvinyl butyral matrices combined with pH indicators and plasticizers for optical waveguide-based ammonia detection [14]. These studies show the significant impact of the polymer matrix and additives on the sensing performance of colorimetric sensor materials, but do not systematically investigate and discuss the impact of the polymer’s properties or structure on the sensing performance of the sensor dye. Furthermore, crucial parameters for the selection of the sensor matrix in order to build up a colorimetric gas sensor, like response and recovery time, and the stability of the sensor material, are not mentioned.

**Figure 1:**
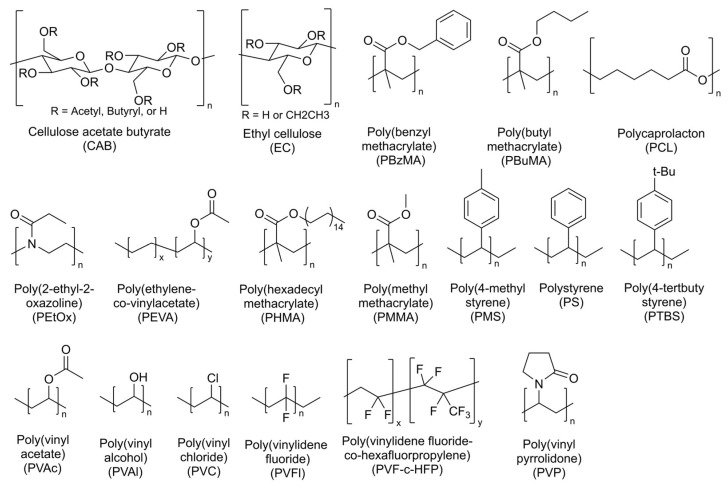
Structure of the investigated polymers as sensor matrix.

In this study, we chose a different approach to focus on the systematic investigation of the influence of polymers on the sensing performance. Specifically, we chose to examine polymers with different properties and structures, which are displayed in Figure 1. The selected polymers differ in polarity, ranging from hydrophilic polymers such as polyvinyl alcohol to lipophilic polymers, such as polystyrene. Furthermore, polymers exhibiting different structural features, such as a heteroatom backbone (e.g., ethyl cellulose and poly(2-ethyl-2-oxazoline)), were investigated. Additionally, we evaluated the effects of steric demands by comparing polymers with identical functional groups but different substituents (e.g., polystyrene, poly(4-methylstyrene), and poly(4-tert-butylstyrene)). To compare the response and recovery times of different polymers, a similar layer thickness was used for the examined materials.

**Figure 2:**
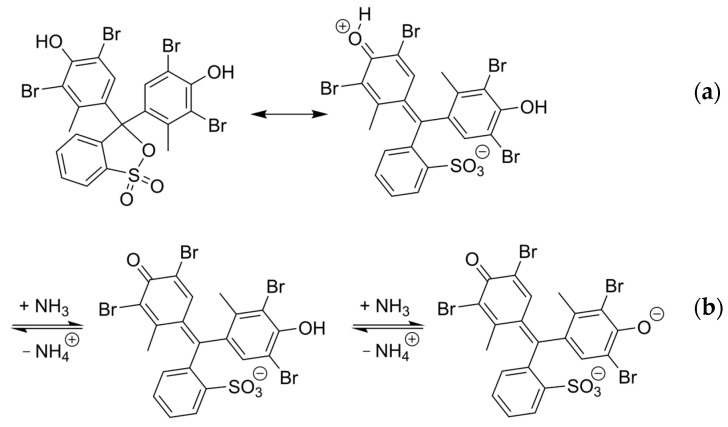
(**a**) Closed and open neutral form of BCG, (**b**) chemical reaction of BCG with ammonia.

This study systematically investigates how the polymer matrix affects the response of colorimetric sensor materials by detecting ammonia as a model analyte. Ammonia is a well-studied analyte, since it is a toxic compound that requires monitoring in several applications such as agriculture and food safety. Various types of gas sensors have been documented for ammonia monitoring, including electrochemical, metal oxide-based, resistance-based, and optical sensors [17,18,19,20,21,22,23,24,25]. Among optical sensors, colorimetric sensors are frequently referenced in the literature for ammonia detection, since they are sensitive, cost-effective, and can be operated at room temperature [25,26]. pH indicators are commonly used as sensor dyes for this purpose. In this study, the pH indicator bromocresol green (BCG) is employed as the sensor dye for ammonia detection, since it is commonly applied as sensor dye for the detection of ammonia [27,28,29,30]. BCG can exist in a closed and open neutral form (Figure 2a) [31]. Ammonia induces a pH-driven reaction in BCG, causing it to transition from its neutral to its dianionic form, resulting in a color change from yellow to blue (Figure 2b). This transformation corresponds to a shift in the absorption maximum from 444 nm to 616 nm in aqueous solution [32].

For the systematic screening of polymers as sensor matrix materials, we developed a camera-based measurement setup for colorimetry. The color camera is applied as a detector and the measurement data are obtained via the color from specific pixel areas of the picture. The color model that is applied for the readout is the RGB (Red, Green, Blue) model. The RGB model is an additive color model, and defines colors through the intensity levels of the colors red, green, and blue. The readout of the RGB values of the sensor materials is commonly applied in colorimetric gas sensing, and able to detect various target gases [33,34,35,36,37]. In this setup, a Raspberry Pi camera is applied to analyze several sensor materials in parallel within a custom-designed measurement chamber. An automated readout is performed using a Python 3 script to analyze changes in the RGB values corresponding to the colorimetric response.

Key parameters for evaluating the sensor material’s performance include sensitivity to the analyte, response and recovery times, and long-term stability. We also investigate the impact of polymers as sensor matrices on cross-sensitivity to environmental factors such as temperature and humidity. This study aims to provide a systematic understanding of the relationship between polymer properties and the sensing performance of colorimetric sensor materials. By investigating polymers with varying structures and functionalities, we aim to compare the sensor materials regarding sensitivity and stability. Additionally, the results serve as a practical guide for the selection of appropriate polymers for colorimetric sensor materials across different applications for ammonia detection.

## 2. Materials and Methods

### 2.1. Preparation of the Sensor Material Films

#### 2.1.1. Reagents

The polymers and the suppliers of the polymers are listed in Table 1. For the dilution of the polymer, the solvents toluene, dichloromethane (DCM), chloroform, tetrahydrofuran (THF), and dimethylformamide (DMF) were purchased from Carl Roth GmbH, Karlsruhe, Germany. Deionized water was obtained from a Millipore Milli-Q water purification system (Millipore Ireland B.V., Cork, Ireland). The dye BCG was purchased from Carl Roth GmbH, Karlsruhe, Germany. All chemicals were used without purification.

#### 2.1.2. Processing and Deposition of the Sensor Material

For the preparation of the sensor material, polymer and sensor dye solutions were produced. All polymers were diluted in a proper solvent, according to Table 1, with a concentration of 0.05 g/mL solution. The sensor dye solution was prepared by dissolving BCG in ethanol in a concentration of 30 mmol/L. A solution of the sensor material was prepared by mixing the polymer and sensor dye solutions in a 4/1 volume ratio.

To prepare thin sensor material films, the sensor material solution was spin-coated on a 1 cm × 1 cm quartz glass piece. To achieve comparable layer thicknesses, different spin speeds varying from 2000 rpm to 10,000 rpm were used according to the rheological behavior of each sensor material solution. The layer thickness of the sensor material films was measured using profilometry (Dektak 8, Veeco, Plainview, NY, USA), and determined to lie between 400 nm and 700 nm. The surface structure was controlled via optical microscopy (DM6 M, Leica, Wetzlar, Germany). The transmission of the sensor materials was measured via UV/Vis spectroscopy (Lambda 25 UV/Vis spectrum, Perkin Elmer, Waltham, MA, USA).

### 2.2. Laboratory Setup for Testing the Sensor Probes and Test Method

#### 2.2.1. Calibration Gas Generator

**Figure 3:**
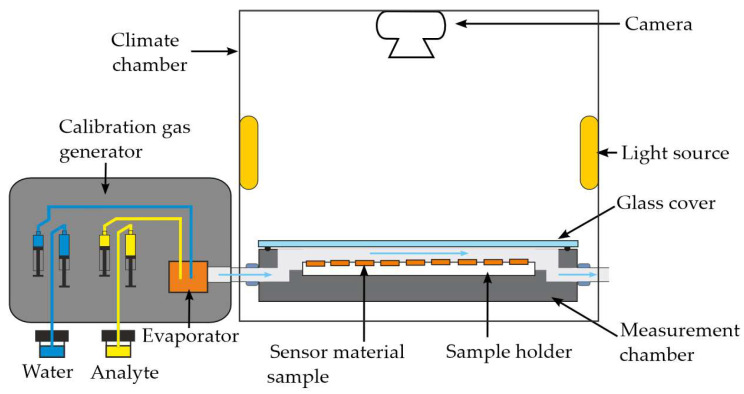
Schematic drawing of the gas measurement chamber.

The analyte gas ammonia was generated using the calibration gas generator (HovaCAL^®^ 7836-VOC, IAS GmbH, Oberursel, Germany) in the respective concentrations of 2.0 ppm to 20 ppm, as illustrated in Figure 3. The calibration gas generator incorporated three distinct manufacturing techniques: continuous syringe injection, capillary dosing, and thermal mass flow control, in accordance with the DIN EN ISO 6145-1:2020-02 standard (ISO) [38], to produce test gases at the desired concentrations. Thereby, the calibration gas generator dosed certain amounts of the 0.1 N aqueous ammonium hydroxide solution between 0.85 and 50 μL/min via syringe pumps over a capillary into an evaporator. Analogous, the test gas was humidified with an accuracy of ±1% r.h. by dosing deionized water using 200 µL syringe pumps over a capillary into an evaporator. The syringe pumps operate in a push–pull mode, meaning that while one syringe dispenses, the other syringe is filled. The automatic switching between the syringes through a dead-volume-minimized rotary valve enables nearly continuous dosing. The evaporator was constantly heated to 75 °C. The evaporated components were instantly diluted with 1500 mL/min of synthetic air to generate certain test gas concentrations. The synthetic air gas flow was regulated by the thermal mass flow controller (MFC). The synthetic air was generated by the synthetic air generator (CG15L, PEAK Scientific Instruments Ltd., Inchinnan, UK). The amount of the liquid component was calculated by applying the ideal gas equation and the standard conditions of 1013.25 mbar and 0 °C. To avoid concentration errors caused by condensation effects in the calibration gas generator, all lines inside the calibration gas generator carrying the test gases were heated to 100 °C. The flow rate of 1000 mL/min was defined by the calibration gas generator.

#### 2.2.2. Gas Measurement Chamber

The gas measurement chamber consists of a milled aluminum housing with a polytetrafluoroethylene (PTFE) multi-sample holder and a gas-tight float glass cover. The total internal volume of the chamber is approximately 80 mL, with dimensions of 180 mm in length, 100 mm in width, and 4 mm in height. The gas measurement chamber is connected to the calibration gas generator via heated pipes made out of PTFE (see Figure 3). The PTFE sample holder takes up to 56 samples for simultaneous measurements and is positioned at the center of the gas measurement chamber. The samples are put into 1 mm-deep recesses arranged in a 9 × 6 grid. The entire setup is placed inside a climate chamber (VMT 04/35, Heraeus Vötsch, Balingen, Germany) to control the temperature during the measurement. The time needed for one complete gas exchange was measured to be below 5 s (Figure S1).

#### 2.2.3. Colorimetric Characterization

The screening of sensor materials was performed by recording an image series upon exposure to the analyte gas ammonia, and analyzing the color changes by determining the RGB values of the sensor materials. The experimental setup consisted of a gas measurement chamber, a camera (12 MP High Quality Camera, Raspberry Pi Foundation, Cambridge, UK), and a white light source (Lumis Slim LED S Bi Color, Rollei, Norderstedt, Germany) with a light temperature of 5500 K. The spectrum of the light source (Figure S2) and spectral sensitivity characteristics of the camera are provided in the Supporting Information (Figure S3). The camera was positioned horizontally above the gas measurement chamber to capture images of the sensor materials. A series of images were recorded at 10 s intervals, with the camera controlled by a single-board computer (Raspberry Pi 4B, Raspberry Pi Foundation, Cambridge, UK).

**Figure 4:**
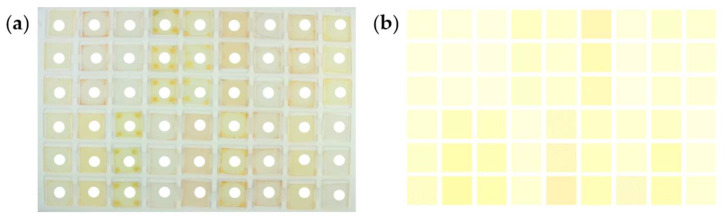
Raw image data recorded by the camera including the superimposed 9 × 6 grid (**a**) and readout of the RGB values averaged over a circular selected region (**b**).

The raw image data, stored in JPG format, were processed using a custom Python 3 script to extract the RGB values. A 9 × 6 grid was superimposed on each image (Figure 4a), and the RGB values were averaged over a circular region with a diameter of 20 pixels at the center of each sensor material (Figure 4b). To ensure reproducibility, three samples of each polymer were measured. The sensor response for each polymer was calculated as the average of the three samples.

## 3. Results

### 3.1. Shifting of Absorption Maxima

The UV/Vis transmission spectrum of the sensor material is recorded via UV/Vis spectrometry. The wavelengths of the absorption maximum λ*_max_* of the protonated and deprotonated forms of BCG are listed in Table 2. The position of λ*_max_* of BCG changes when integrated into different sensor matrices. According to the literature, the protonated form of BCG in an aqueous environment has a λ*_max_* of 437 nm, but when integrated into a polymer, the λ*_max_* shifts up to 22 nm [39]. It is known that the integration of a pH-sensitive dye into a polymer matrix affects its spectral properties, leading to a shift in λ*_max_*. The λ*_max_* is determined by the energy gap between the highest occupied molecular orbital (HOMO) and the lowest unoccupied molecular orbital (LUMO). A larger energy gap between the HOMO and LUMO results in a lower λ*_max_*. This phenomenon is related to the solvatochromism of the dye. Solvatochromism is caused by different solvation effects on the ground and the first excited states of the dye molecule. If the dye is better stabilized in its excited state than in its ground state, a bathochromic shift occurs, also called positive solvatochromism. Kassal et al. concluded that the excited state of the protonated form of BCG is more polar than the ground state, and therefore exhibits a positive solvatochromism [40]. The location of λ*_max_* of the protonated form is an indication of the polarity of the matrix. Thus, sensor materials with a higher λ*_max_* are assumed to be more polar.

The exception is the polymer PHMA, which has a significant shift in λ*_max_* compared to the other polymers. This can be explained by the aggregation of the dye within the PHMA matrix, which can be observed in the microscope images. For the sensor materials containing the polymers PEVA, PVC, PVFl, and PVF-c-HF, no λ*_max_* could be determined due to a broad absorption band.

### 3.2. Response Towards Ammonia

**Figure 5:**
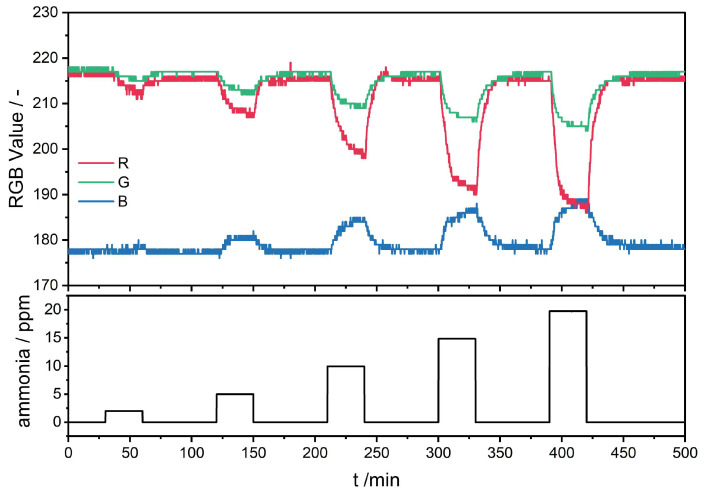
Sensor response of the PCL/BCG sensor material at 25 °C and 50% r.h. upon exposure to 2.0, 5.0, 10, 15, and 20 ppm of ammonia.

The sensor material reacts to ammonia via color changes determined by the R, G, and B values. The pixels of the camera chip have the highest spectral sensitivity (>70%) for the B value from 415 to 480 nm, for the G value from 480 to 580 nm, and for the R value from 590 to 660 nm. λ*_max_* of the protonated form of the sensor materials is between 415 and 452 nm, and the deprotonated form between 610 nm and 636 nm. Therefore, the pixels for the R and B values are in line with λ*_max_*, and are expected to show the highest intensity changes. With an increasing ammonia concentration, the color of BCG changes from yellow to blue. Due to the higher absorption of light in the R region upon ammonia exposure, the R value decreases, as shown exemplarily in Figure 5 for the sensor material PCL/BCG.

The B value increases with rising ammonia concentrations, due to the decreasing intensity at λ*_max_* of the protonated form of BCG, which aligns with the literature [39]. Since the change in the R and B values are proportional, for comparing the impact of the polymers, the R value is used in the following sections for the comparison of the sensor materials.

### 3.3. Sensitivity

**Figure 6:**
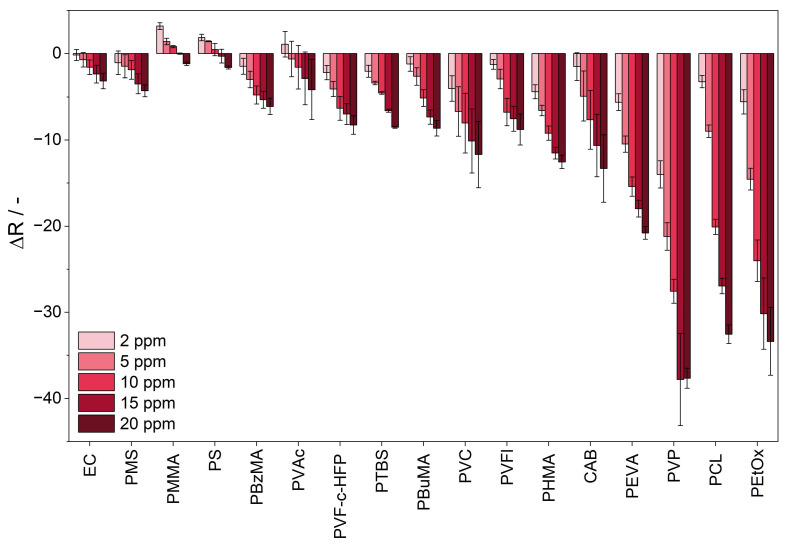
Response of the R value of the sensor materials upon exposure to 2.0, 5.0, 10, 15, and 20 ppm of ammonia at 25 °C and 50% r.h. The sensor materials are labeled by their sensor matrix component and arranged in order of increasing sensitivity.

The sensitivity of the sensor materials and the impact of the polymer sensor matrix are determined via the difference between the R value upon ammonia exposure (ΔR). This is calculated based on the difference between the mean of five minutes before dosing and the last five minutes of the ammonia dosing. Sensitivity is defined as the slope of the calibration curve of the response to ammonia. The response of the sensor material upon exposure to ammonia is displayed in Figure 6. The sensor material PVAl/BCG is not displayed in Figure 6, as the sensitivity could not be determined, since the sensor response of the material does not reach a plateau within 30 min of ammonia exposure. Figure 6 shows that the extent of the color reaction to ammonia is influenced by the sensor matrix. Sensor materials containing the polymers PEVA, PVP, PCL, and PEtOx show the highest sensitivity upon ammonia exposure, while sensor materials containing PMMA, PS, and EC exhibit a significantly lower sensitivity to ammonia. The sensitivity is determined by the slope of the ΔR plot against the ammonia concentration.

In this experimental study, the sensor material PEtOx/BCG is the most sensitive sensor material upon exposure to ammonia. PVP and PEtOx are both amide-based, hydrophilic polymers. Hydrophilic polymers are defined as polymers that dissolve or swell in water [41]. It is reported that polar gases can interact with polar polymers and show a higher permeability in polar polymers [42]. Thus, the polar environment provided by the hydrophilic polymers might lead to an increase in the permeability of ammonia, since ammonia is a polar, basic gas. A high gas permeability of the sensor matrix is advantageous for the detection of gaseous analytes. The higher the permeability of the sensor matrix, the more analyte diffuses into the sensor matrix and can react with the sensor dye, leading to an increased color reaction.

The sensor material PCL/BCG reveals high sensitivity upon exposure to ammonia. PCL is a crystalline aliphatic polyester composed of hexanoate repeat units. It is reported to have high water and gas permeability [43,44,45]. This can be assigned to its low glass transition temperature (−60 °C) in comparison to the polymers investigated in this study (from −35 to 130 °C). A lower glass transition temperature results in higher chain mobility, which improves the diffusion and solubility of gases. The increase in free volume associated with the glass transition temperature further enhances the permeability of gases [46].

Accordingly, the reduced sensitivity of sensor materials with hydrophobic polymers, like PMMA, PS, and EC, as sensor matrices can be attributed to the lower solubility of ammonia in these hydrophobic polymers.

Furthermore, the length of the polymer’s side chain affects the sensitivity of the sensor material towards the analyte. Among the sensor materials containing poly (alkyl methacrylate) polymers, PMMA/BCG demonstrates the lowest sensitivity to ammonia. Sensor materials with the same main chain, but increasing side chain length, like PBzMA/BCG and PBuMA/BCG, do show a significantly higher response upon ammonia exposure. PHMA/BCG, which features the longest side chain, exhibits the most significant response upon ammonia exposure. This might be related to the increase in permeability to ammonia. Kawakami et al. found out that for oligodimethyl siloxanyl-substituted polymers, the longer the siloxane side chain, the higher the diffusion coefficient in gas permeation for oxygen [47]. Since the permeability of the analyte is directly related to the diffusion, high permeability of the analyte into the sensor matrix might cause high sensitivity. Furthermore, Mogri et al. confirmed that the side chain length for poly (alkyl acrylates) significantly affects their gas permeability [48]. Accordingly, sensor materials composed of the styrene polymers PS and PMS show a limited reactivity to ammonia. However, the incorporation of a t-butyl side chain in PTBS enhances sensitivity toward ammonia. It has a greater steric demand and might increase the permeability of ammonia into the sensor material.

When comparing the sensitivity of a homopolymer and its corresponding copolymers, no general trend is observed. It depends upon the impact of the copolymer on the polymer properties in comparison to the homopolymer. In the case of the homopolymer PVFl and the copolymer PVF-c-HFP, the sensor materials show similar sensitivity upon ammonia exposure. Both are fluorinated polymers and show similar properties regarding solubility and polarity [49]. In contrast, the polymer PVAc and the corresponding copolymer PEVA both contain a vinyl acetate main chain. The sensitivity of sensor materials containing the copolymer PEVA is significantly higher than for PVAc. Due to the integration of polyethylene into PEVA, the properties of the polymers, such as the solubility, change significantly [50]. Furthermore, the water and gas permeability of PEVA is affected by the content of polyvinyl acetate in PEVA [51].

### 3.4. Response and Recovery Time

The response and recovery times of the sensor materials are displayed in Table 3. The response time is defined as the time taken for the sensor response to increase from 10% (t10) to 90% (t90) during analyte dosing. Conversely, the recovery time is defined as the time taken for the sensor response to decrease from t90 to t10 after analyte dosage. As Table 3 shows, the response and recovery times are affected by the choice of sensor matrix. They vary from several minutes for the EC to more than 30 min for the PVAl-based sensor materials. The response times of PMMA/BCG and PS/BCG could not be determined (n.d.), since the response upon ammonia is not significant at 20 ppm.

For all sensor materials, the response time is faster than the recovery time. The sensitivity of the sensor material is correlated to the response and recovery time. The most insensitive sensor material shows the fastest response. In general, sensor materials with a high sensitivity to ammonia, e.g., PVP/BCG, PCL/BCG, and PEtOx/BCG, also reveal slower response and recovery times. The reaction of ammonia with BCG is a gas–solid phase reaction. It initially includes the adsorption of analyte molecules onto the surface of the sensor material, followed by the diffusion of these molecules within the sensor matrix and the chemical reaction between the analyte and the sensor dye. Therefore, the response and recovery times are affected by the reaction kinetics of ammonia with BCG, and the diffusion into the sensor matrix.

Notably, PVAl/BCG exhibited the slowest response, where the signal did not reach saturation, indicating that a plateau was not achieved within the 30 min testing period. This slow response may be attributed to the presence of hydroxyl groups in the polymer structure, which can interact with ammonia, potentially delaying the response to ammonia [52].

### 3.5. Influence of Humidity

**Figure 7:**
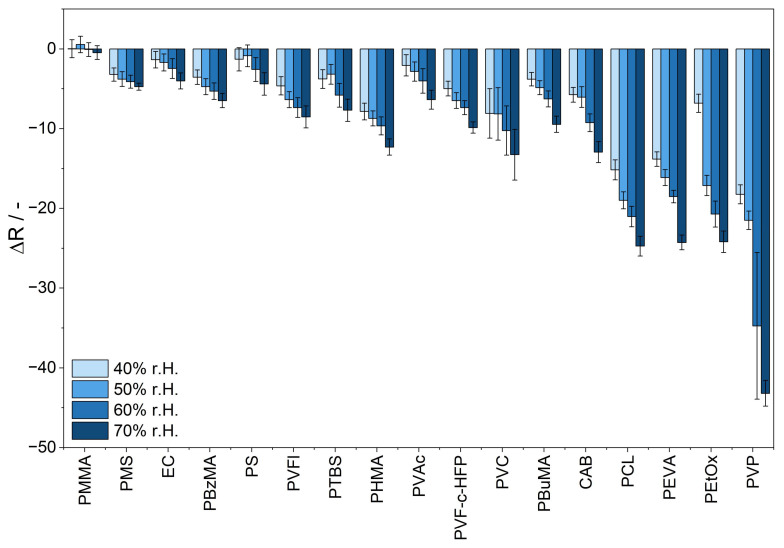
Response of the R value of the sensor materials upon exposure to 10 ppm of ammonia at 25 °C and humidity levels ranging from 40% to 70% r.h. The sensor materials are labeled by their sensor matrix component and arranged in order of increasing sensitivity.

The influence of humidity on the response to ammonia of each sensor material is investigated by measuring the response to 10 ppm of ammonia at alternating humidity levels ranging from 40% to 70% r.h. at 25 °C. As Figure 7 demonstrates, the influence of humidity on the sensitivity of the sensor material is affected by the choice of the polymer matrix. The sensor materials PVP/BCG and PEtOx/BCG exhibit the highest signal change upon 10 ppm of ammonia with alternating levels of humidity.

A low influence of humidity is shown by sensor materials containing PMMA, PMS, and EC. With an increase in humidity, the signal amplitude upon 10 ppm of ammonia exposure remains within the measurement uncertainty range, and therefore the polymers are not significantly influenced by humidity.

There are several factors explaining the impact of humidity on colorimetric sensor materials with polymers as sensor matrices, as summarized by Yu et al. [53]. Water may react with ammonia to form ammonium hydroxide [15]. Thus, the presence of water emphasizes the acid-based reaction between ammonia and BCG, resulting in a more pronounced color change and increased sensitivity. It is also reported that the penetration of water vapor into the hydrophobic plastic film of a solid-state colorimetric sensor can lead to plasticization, enhancing polymer chain mobility and increasing sensitivity.

Through the selection of the sensor matrix, the cross-sensitivity to humidity can be tuned and the humidity interference can be mitigated. Specifically, the hydrophilic sensor materials composed of PVP and PEtOx demonstrate a significant change in signal intensity with increasing humidity levels. The hydrophilic character of these polymers might facilitate the uptake of water into the matrix. Furthermore, the sensor matrices that are reported to have a high water permeability, such as PCL and PEVA, show a comparably higher cross-sensitivity to humidity. In contrast, hydrophobic sensor matrices, such as PMMA, PMS, and EC, lead to lower cross-sensitivity to humidity. Due to the hydrophobic character of the matrix, the penetration of water into the sensor matrix might be reduced.

### 3.6. Influence of Temperature

**Figure 8:**
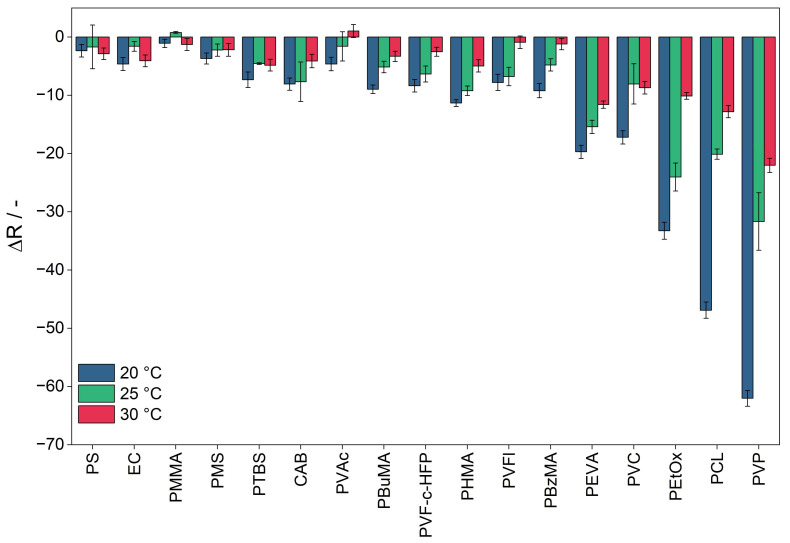
Response of the R value of the sensor materials upon exposure to 10 ppm of ammonia at 20 °C, 25 °C, and 30 °C at 50% r.h. The sensor materials are labeled by their sensor matrix component and arranged in order of increasing sensitivity.

The influence of temperature is determined by the response of the sensor materials to 10 ppm of ammonia at alternating temperatures (20, 25, and 30 °C). To exclude the humidity effects, the absolute humidity levels at each temperature level were adjusted to remain at a constant relative humidity level of 50% during this experiment. The sensor materials exhibit cross-sensitivity to temperature, indicating that their response to ammonia is influenced by temperature variations (Figure 8). Generally, the sensor response decreases as the temperature increases. Notably, there is a trend where more sensitive materials demonstrate a higher sensitivity to temperature.

The response of the sensor materials is affected by several factors, including the solubility and diffusion of the analyte within the sensor material, as well as the reaction between the analyte and BCG. Among these factors, the predominant influence is from the exothermic reaction between BCG and the analyte. As the temperature rises, the equilibrium of this reaction shifts to the reactants, resulting in a lower sensor response to ammonia at elevated temperatures.

While it is expected that changes in diffusion and solubility will impact the sensor response, these factors do not appear to have a significant effect in this context. Although solubility and diffusion generally increase with rising temperatures, their contribution to the overall sensor response is minimal compared to the effects of the reaction dynamics.

### 3.7. Stability of the Sensor Material

**Figure 9:**
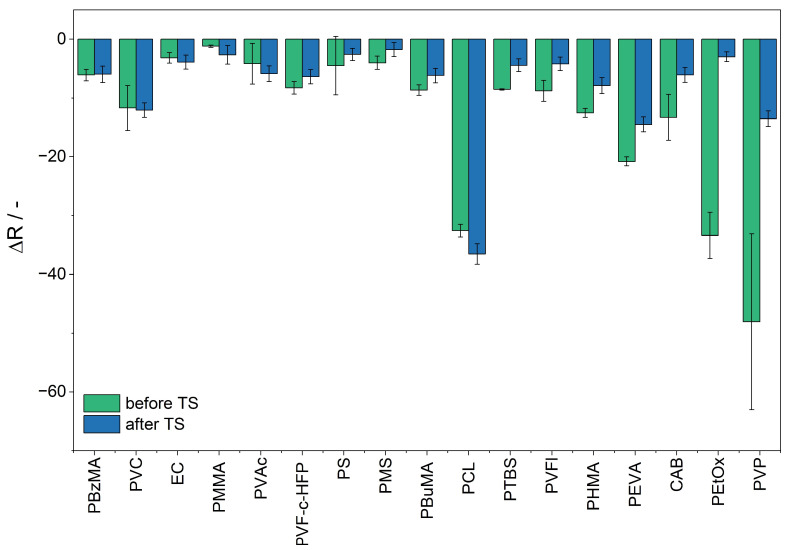
Response of the R value of the sensor materials upon exposure to 20 ppm of ammonia at 25 °C before and after exposure to temperature stress. The sensor materials are labeled by their sensor matrix component and arranged in order of increasing stability.

The stability of sensor materials is crucial for their performance in various applications. This study examines the effects of temperature stress on different polymers used as sensor matrices (Figure 9). The sensor materials were subjected to a temperature of 40 °C with 50% r.h. in the presence of 30 ppm of ammonia added every 2 h for a duration of 30 min over a period of 160 h in order to increase polymer aging.

To assess the impact of increased-temperature aging, the testing temperature was set to 40 °C, which is 20 °C higher than the standard temperature, yet below the melting point of the materials. The results indicate that certain polymers exhibit stability, showing no significant change in signal post exposure. Stable sensor materials include PBzMA/BCG, EC/BCG, and PVC/BCG.

**Figure 10:**
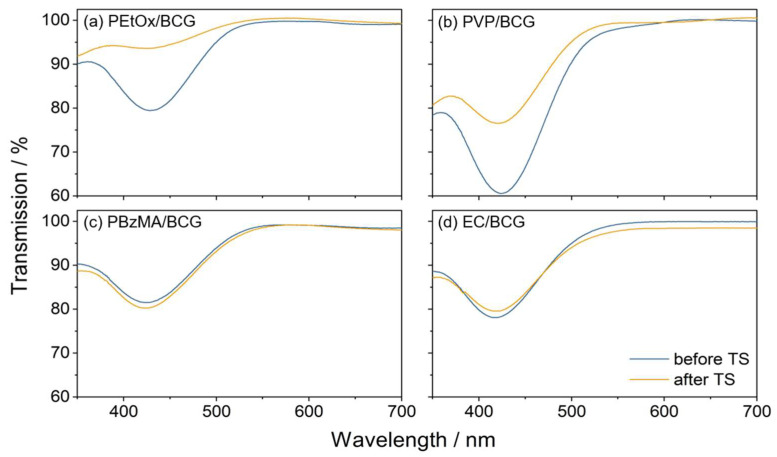
UV/Vis spectra of (**a**) PEtOx/BCG, (**b**) PVP/BCG, (**c**) PBzMA/BCG, and (**d**) EC/BCG recorded before and after temperature stress (TS).

In terms of UV-Vis transmission, the examination revealed no significant changes before or after temperature stress for these stable polymers (Figure 10c,d). Conversely, a decrease in response was observed in several sensor materials consisting of the polymers PVP, PEtOx, PEVA, CAB, PTBS, PMS, PS, PBuMA, PHMA, PVFl, and PF-c-HFP. An increase in response was noted for PMMA/BCG, PVAc/BCG, and PCL/BCG.

Additionally, sensor materials, such as PVP/BCG, PEtOx/BCG, PEVA/BCG, and PHMA/BCG, exhibited increased transparency measured via UV-Vis spectroscopy (Figure 10a,b). A smaller decrease in absorption was noted for PCL/BCG, PMMA/BCG, PBuMA/BCG, PVAc/BCG, and PVFl/BCG. A small increase in absorption was observed for PS/BCG, while PMS/BCG showed no change in absorption. Sensor materials containing polymers with high ammonia sensitivity exhibit, in general, reduced stability. The alterations in sensitivity may be attributed to various factors. The aggregation or leaching of the dye can contribute to a decrease in stability. The dye is physically trapped within the polymer and is capable of leaching or migrating through it. This may explain the observed increase in transmission in the UV/Vis measurements of the sensor materials, such as PVP/BCG and PEtOx/BCG [54]. Furthermore, the degeneration of the polymer matrix can lead to a change in ammonia sensitivity. Environmental factors such as heat, UV light, ozone, oxygen, and humidity accelerate polymer aging [55]. For instance, it has been reported that polymers, e.g., PCL, undergo degradation at elevated temperatures. Therefore, weight loss can occur, as well as a change in mechanical properties, e.g., porosity and brittleness [56]. This might lead to a change in the physical properties of the polymer matrix, like the morphology or density. Therefore, the permeability of the analyte into the sensor material might be affected, and the sensitivity of the sensor material changes.

## 4. Discussion

The results of this study on the impact of polymers as matrix materials are summarized in Table 4. To compare the influence of different polymer matrices on key parameters—sensitivity, response time, recovery time, humidity interference, temperature interference, and stability—the values are normalized on a scale from 0 to 100. A score of 100 represents the most advantageous properties: the highest sensitivity, fastest response and recovery times, lowest interference with humidity and temperature, and highest stability compared to other sensor matrices. Conversely, a score of 0 corresponds to the least favorable properties: the lowest sensitivity, slowest response and recovery times, highest interference with humidity and temperature, and lowest stability.

Sensitivity, humidity, and temperature interference are evaluated by determining the slope of the ΔR plot against the respective parameter, with normalization across the polymers on a 0 to 100 scale. The response and recovery times are directly normalized based on their measured values. Stability is assessed by comparing the sensor’s response before and after temperature stress, with the difference normalized accordingly.

The sensor response of the sensor materials to ammonia is significantly influenced by the properties of the sensor matrix. In general, sensor materials that show high sensitivity to ammonia also show a high cross-sensitivity to humidity and temperature, and reduced stability. Sensor matrices, which emphasize the response of the sensor dye BCG to ammonia, are hydrophilic, like PEtOx and PVP, or reveal a high permeability into the analyte, like PCL and PEVA. In contrast, sensor materials that are less sensitive to ammonia are also less sensitive to humidity and temperature and display increased stability. Examples of polymer matrices that lead to the low sensitivity to ammonia of the sensor matrix are the hydrophobic polymers EC, PMS, and PMMA. Sensor matrices that are hydrophobic, but incorporate side chains with high steric demands, such as PTBS and PHMA, do lead to an increase in sensitivity compared to less polar materials lacking such structural features.

## 5. Conclusions

This study demonstrates the significant impact of polymers as matrix materials on colorimetric sensor materials, exemplified by the detection of ammonia. The camera setup enables the simultaneous analysis of the sensor materials. It is suitable to investigate and compare the effects of polymers as sensor matrices. The choice of sensor matrix plays a crucial role in sensitivity to the analyte, response and recovery time, cross-sensitivity to environmental factors such as temperature and humidity, and the overall stability. Understanding these relationships is essential for the optimization of the gas sensor performance and reliability of sensor materials. Therefore, it is important to consider the matrix material when designing a colorimetric sensor. The choice of sensor matrix depends upon the application of the sensor. The findings provide a valuable overview that can serve as guidance for selecting polymer sensor matrices for the detection of ammonia. For instance, sensor materials with hydrophilic polymers like PVP and PEtOx exhibit high sensitivity in this study, but are considered unsuitable for the long-term monitoring of ammonia due to their low stability. Suitable sensor matrices for this application might be more stable polymer matrices like PBzMA and PVC, since these polymers show a significant response to ammonia, and high stability. For application as a sensor material to detect ammonia, the sensitivity of these sensor materials could be increased by the addition of additives like plasticizers, or the choice of the sensor dye. For instance, a pH indicator with a lower pKa value than BCG (e.g., bromophenol blue) can lead to higher sensitivity upon exposure to ammonia [14]. Furthermore, for the sensor application, the response time could be optimized by reducing the layer thickness.

## Figures and Tables

**Table 1:** List of polymers applied in this study.

Polymer	Supplier	Molar Mass	Solvent
Ethyl cellulose	Sigma-Aldrich	cp 10	Toluene
Cellulose acetate butyrate	Sigma-Aldrich	30,000	DCM
Polystyrene	Carl Roth	320,000	Toluene
Polycaprolactone	Sigma-Aldrich	80,000	DCM
Poly (4-methylstyrene)	Sigma-Aldrich	72,000	Toluene
Poly (4-tert-buthylstyrene)	Sigma-Aldrich	50,000–100,000	Toluene
Poly methyl methacrylate	Sigma-Aldrich	350,000	Toluene
Poly (benzyl methacrylate)	Sigma-Aldrich	100,000	Toluene
Poly (butyl methacrylate)	Sigma-Aldrich	337,000	Toluene
Poly (hexadecyl methacrylate)	Sigma-Aldrich	200,000	Toluene
Polyvinyl acetate	Carl Roth	55,000–70,000	Toluene
Poly (ethylene-co-vinyl acetate)	Sigma-Aldrich	n.a.	DCM
Poly (vinylidene fluoride)	Thermo Fischer	n.a.	DMF
Poly(vinylidene fluoride-co-hexa-fluoropropylene)	Sigma-Aldrich	400,000	DMF
Polyvinyl alcohol	Sigma-Aldrich	160,000	Water
Polyvinyl chloride	Carl Roth	21,000	THF
Polyvinyl pyrrolidone	Carl Roth	900,000–1,200,000	Water
Poly (2-ethyl-2-oxazoline)	Thermo Fischer	200,000	Water

n.a.: not applicable.

**Table 2:** λ*_max_* of the protonated and deprotonated dye BCG integrated into different sensor matrices.

Sensor Matrix	λ*_max_* (Protonated Form)/nm	λ*_max_* (Deprotonated Form)/nm
PVAc	415	617
PMMA	416	610
CAB	419	611
EC	419	628
PS	420	618
PBzMA	424	628
PMS	424	624
PCL	424	618
PBuMA	425	617
PTBS	425	618
PVP	425	629
PVAl	429	627
PEtOx	429	628
PHMA	452	636

**Table 3:** Response and recovery times of the polymer/BCG sensor material upon exposure to 20 ppm of ammonia at 25 °C and 50% r.h.

Sensor Matrix	Response Time/Min	Recovery Time/Min
PMMA	n.d.	n.d.
PS	n.d.	n.d.
EC	2.5	11.5
PMS	2.7	14.1
PBzMA	2.8	13.6
PVF-c-HFP	3.8	19.4
PVFl	3.8	23.4
PVAc	4.2	10.6
PTBS	5.5	32.2
PHMA	5.7	24.1
PBuMA	6.0	25.1
CAB	6.8	23.4
PEVA	7.5	26.3
PVC	8.0	24.1
PVP	8.0	31.7
PCL	10.5	16.8
PEtOx	12.0	17.7
PVAl	>30	62.3

n.d.: not determined.

**Table 4:** Summary of the results of the polymer study. The parameters are normalized from 0 to 100, where 100 represents the advantageous property.

Polymer	Sensitivity	Response Time	Regeneration Time	Humidity	Temperature	Stability
EC	0	100	96	92	98	98
PMS	2	100	84	97	95	94
PMMA	2	n.d.	n.d.	100	96	96
PS	2	n.d.	n.d.	89	100	95
PBzMA	5	97	86	91	79	100
PVAc	8	83	100	85	85	80
PVF-c-HFP	10	86	59	83	84	95
PTBS	13	69	52	86	94	89
PBuMA	16	63	63	81	84	93
PVC	18	42	36	81	78	99
PHMA	18	67	36	85	84	87
PVFl	19	86	39	87	81	87
CAB	33	54	39	74	89	79
PEVA	40	47	26	62	79	82
PVP	68	42	0	0	0	0
PCL	98	0	71	65	21	89
PEtOx	100	12	66	32	41	12

n.d.: not determined.

## Data Availability

The data presented in this study are available on request from the corresponding author.

## Supplementary Materials

The following supporting information can be downloaded at: https://www.mdpi.com/article/10.3390/s25092829/s1, Figure S1. Gas exchange time within the measurement chamber evaluated using two gas sensors (MiCS5524, Adafruit) mounted on the glass cover at the gas inlet and outlet of the gas measurement chamber. Figure S2. Spectrum of the light source (Rollei LUMIS Slim LED S Bi Color, Germany) at the temperature 5500 K and intensity of 50%. Figure S3. Spectral sensitivity of the Raspberry Pi Camera HQ [57].
