# Supplementary material for: Investigation of Polymers as Matrix Materials for Application in Colorimetric Gas Sensors for the Detection of Ammonia
Source: Sensors (Basel). 2025 Apr 30;25(9):2829. doi: 10.3390/s25092829 (PMC12074213; doi:10.3390/s25092829)
Supplement: Supplementary file 1 [file sensors-25-02829-s001.zip › sensors-3566653-supplementary.pdf]

Supporting Information

Investigation of Polymers as Matrix Materials for the Application in Colorimetric Gas Sensors for the Detection of Ammonia

Sonja Hoffmann [1,2], Michael Henfling [1] and Sabine Trupp [1]

Figure S1

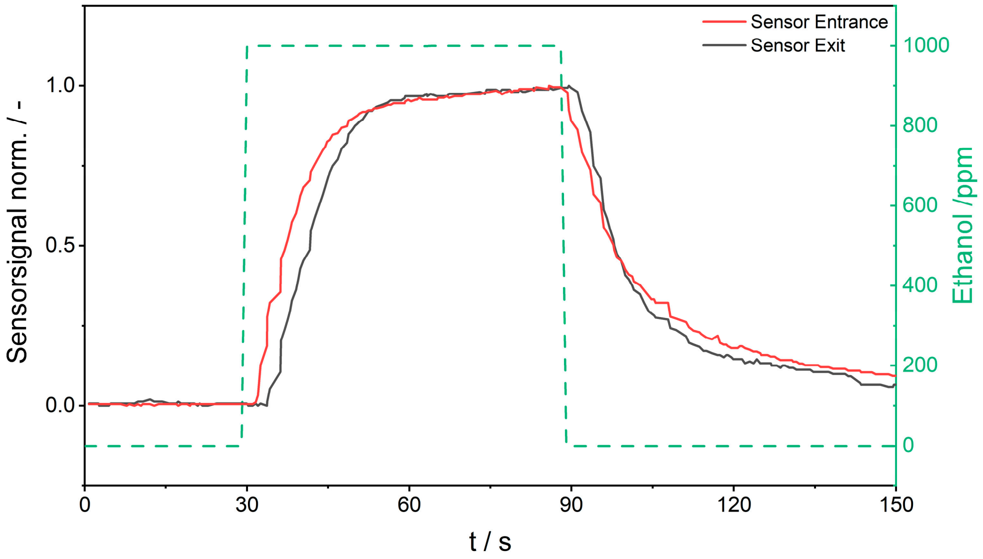

Figure S1. Gas exchange time within the measurement chamber evaluated using two gas sensors (MiCS5524, Adafruit) mounted on the glass cover at the gas inlet and outlet of the gas measurement chamber.

Figure S2

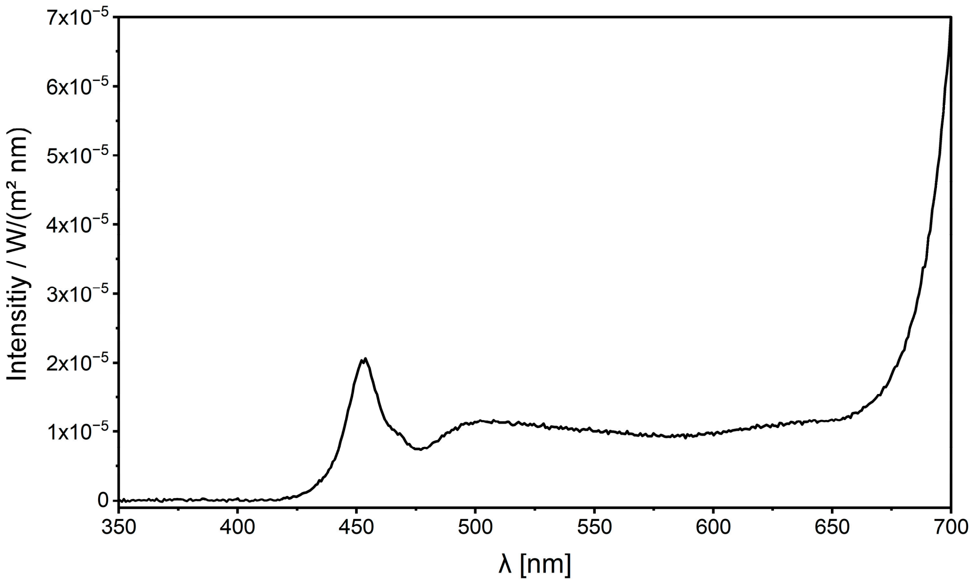

Figure S2. Spectrum of the light source (Rollei LUMIS Slim LED S Bi Color, Germany) at the temperature 5500K and intensity of 50%.

Figure S3

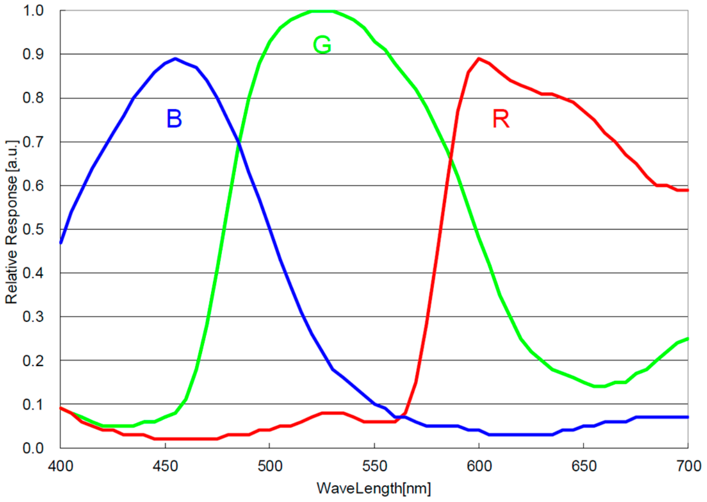

Figure S3. Spectral sensitivity of the Raspberry Pi Camera HQ [57].
